# Hypermobile Anterior Horn of the Lateral Meniscus: A Case Report and Literature Review

**DOI:** 10.1155/2020/8870156

**Published:** 2020-12-23

**Authors:** Hirotaka Nakashima, Yasuhiro Takahara, Yoichiro Uchida, Hisayoshi Kato, Satoru Itani, Yoshitaka Tsujimura, Makoto Nakamura, Yuichi Iwasaki

**Affiliations:** Department of Orthopedic Surgery, Nippon Kokan Fukuyama Hospital, 1844 Tsunoshita Daimon-cho, Fukuyama City, Hiroshima 721-0927, Japan

## Abstract

Hypermobile meniscus is known as one of the causes of knee pain and locking or limitation of the range of motion during knee flexion, even when there is no evidence of meniscus tear on magnetic resonance imaging (MRI). Most such cases show excessive hypermobility of the posterior part of the lateral meniscus. This case report presented a rare case of a hypermobile anterior horn of the lateral meniscus. An 18-year-old woman visited our hospital for left knee pain without trauma. Her physical examination showed a limited range of motion and tenderness in the lateral joint space. However, her MRI did not show any abnormalities. After conservative treatment failed, we performed arthroscopic surgery. The arthroscopic evaluation showed no meniscus and no other intraarticular injury. However, the anterior horn of the lateral meniscus was easily translated beyond the lateral condyle by using a probe. Thus, hypermobile anterior horn of the lateral meniscus was diagnosed. The meniscus was stabilized by the outside-in technique. Immediately after surgery, the catching symptom and pain were alleviated. After three-and-a-half months, she returned to work. The Lysholm score improved from 55, preoperatively, to 100, 1-year postoperatively. In conclusion, careful arthroscopic evaluation is essential for the diagnosis of a hypermobile anterior horn of the lateral meniscus. Arthroscopic meniscus stabilization provides a good outcome for hypermobile meniscus.

## 1. Introduction

The hypermobile meniscus is known as one of the causes of knee pain and locking or limitation of the range of motion (ROM) during knee flexion, even when there is no evidence of meniscus tear in magnetic resonance imaging (MRI) [1–4]. Most cases of hypermobile meniscus show excessive hypermobility of the posterior part of the lateral meniscus [1–4]. Hypermobile lateral meniscus was usually diagnosed by translation beyond the midpoint of the lateral condyle or tibia by using a probe [3, 4]. This case report presents a rare case of a hypermobile anterior horn of the lateral meniscus. To the best of our knowledge, there are no previous reports on the isolated hypermobile anterior horn of the lateral meniscus.

The patient and her family provided informed consent for the publication of this case report.

## 2. Case Report

**Figure 1:**
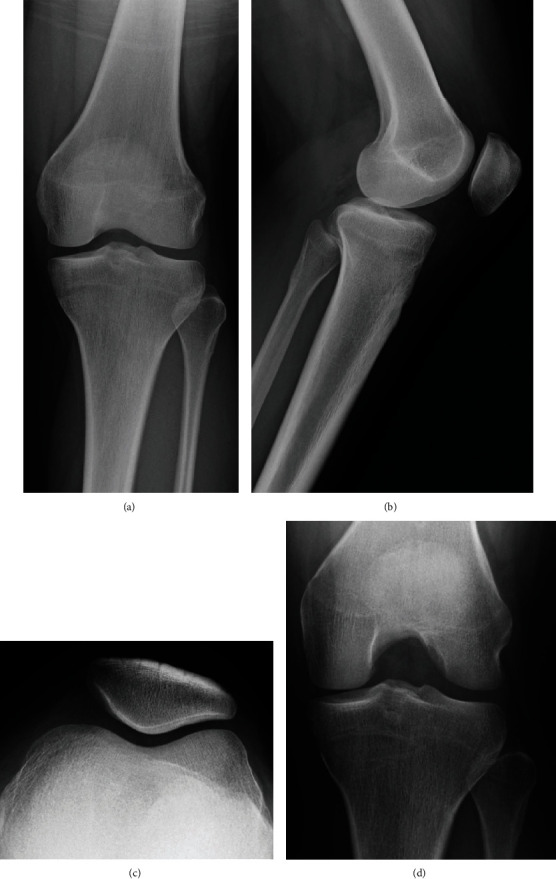
Preoperative radiography. The preoperative radiography was normal. (a) Anteroposterior view; (b) lateral view; (c) skyline view; (d) Rosenberg view.

**Figure 2:**
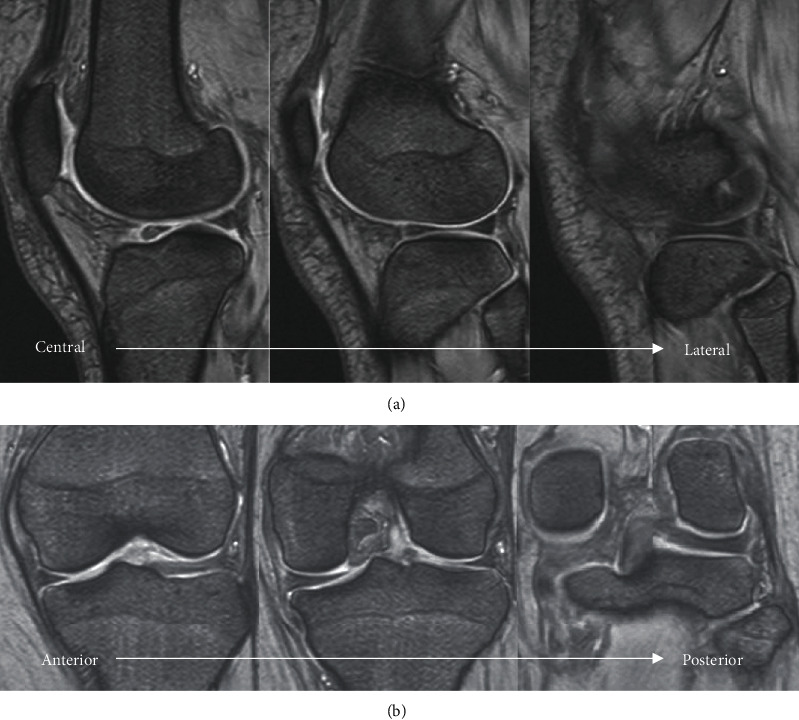
Preoperative MRI. Preoperative MRI showed no abnormal intensity at the meniscus. (a) T2^∗^ sagittal; (b) T2^∗^ coronal. MRI: magnetic resonance imaging.

An 18-year-old woman visited our hospital for left knee pain and a limited range of motion (ROM), without any history of trauma. She had similar symptoms when she was 13-years-old. A physical examination of her left knee showed a limited ROM (extension, -10°; flexion, 100°) and lateral joint tenderness. There was no joint effusion, and Lachman's test was negative. McMurray test was not performed because of knee pain. The Beighton score was 7, which indicated a general joint laxity [5]. Radiographic evaluation was normal, and the MRI did not show any abnormality (Figures 1 and 2).

We started conservative treatment, including rest and physical therapy; however, it was not effective. After the conservative treatment, her physical examination still showed limited ROM (extension, -10°; flexion, 130°), positive lateral joint tenderness, and positive McMurray test. Thus, two months after her first visit, we decided to perform an arthroscopic evaluation and surgery. The preoperative physical examination is shown in Table 1.

**Figure 3:**
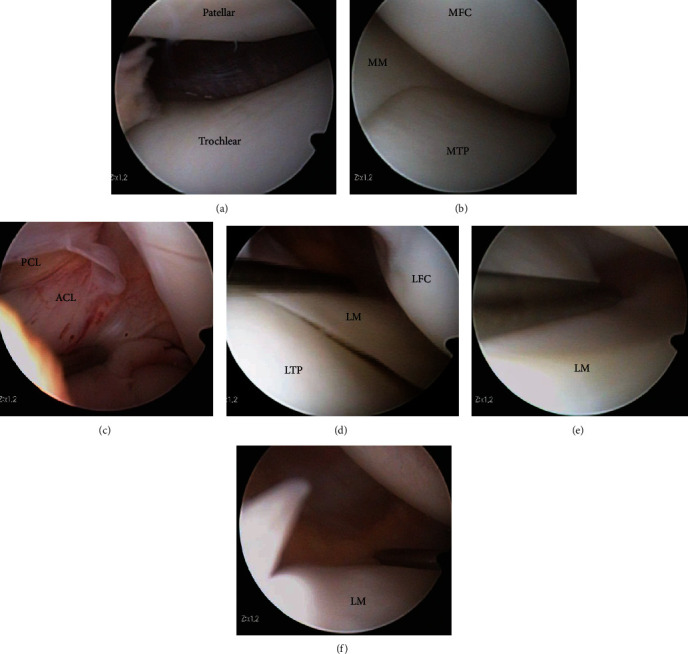
Arthroscopic findings. (a) No cartilage injury at the patellofemoral joint; (b) no meniscus or cartilage injury in the medial compartment; (c) ACL and PCL were intact; (d–f) no posterior lateral meniscus or cartilage injury. Figures 2(d) and 2(e) were viewed from the anterolateral portal.  Figure 2(f) was viewed from the anteromedial portal. MM: medial meniscus; MFC: medial femoral condyle; MTP: medial tibial plateau; ACL: anterior cruciate ligament; PCL: posterior cruciate ligament; LM: lateral meniscus; LFC: lateral femoral condyle; LTP: lateral tibial plateau.

**Figure 4:**
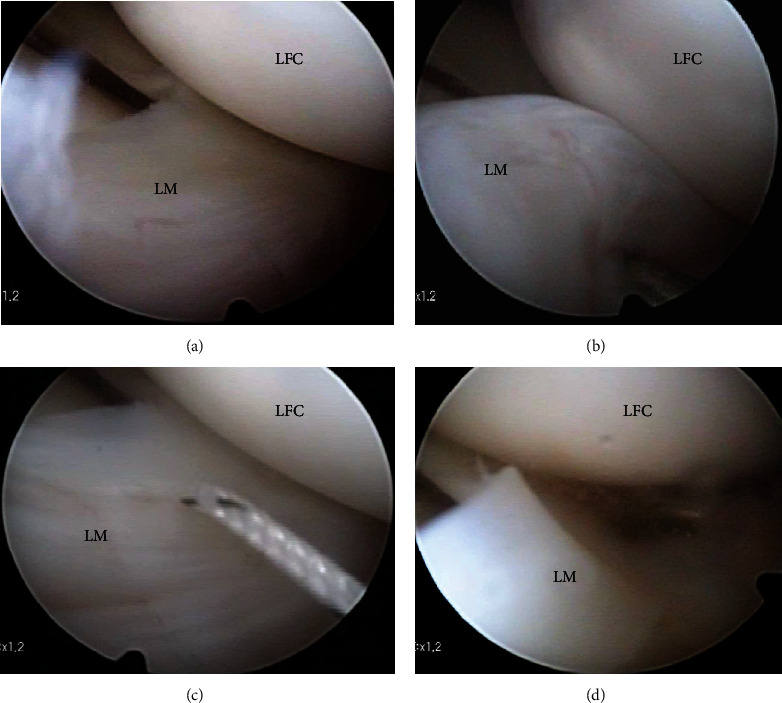
Arthroscopic stabilization of a hypermobile anterior horn of the lateral meniscus. (a) No tear in the anterior lateral meniscus; (b) Evaluation using the probe showed hypermobility at the anterior horn of the lateral meniscus; (c) Meniscus stabilization was performed by the outside-in technique with two stitches using a strong suture; (d) After stabilization, the hypermobility of the anterior horn of the lateral meniscus was resolved. LM: lateral meniscus; LFC: lateral femoral condyle; LTP: lateral tibial plateau.

Arthroscopy showed no abnormal findings in the patellofemoral joint, medial compartment, and intraarticular ligaments (Figures 3(a)–3(c)). The lateral meniscus was not a discoid meniscus. The posterior part of the lateral meniscus showed no tear or hypermobility (Figures 3(d)–3(f)). However, the anterior horn of the lateral meniscus was easily translated beyond the lateral condyle by using a probe (Figures 4(a) and 4(b)). Hypermobile anterior horn of the lateral meniscus was diagnosed, and meniscus stabilization was performed. Meniscus stabilization was performed by the outside-in technique by taking two stitches using a strong suture (Figure 4(c)). After stabilization, the hypermobility of the anterior horn of the lateral meniscus was resolved (Figure 4(d)).

Immediately after surgery, the catching symptom and pain were alleviated. Three months after the surgery, she had no symptoms. Three-and-a-half months after surgery, she could return to work with no physical limitation. The 1-year postoperative physical examination results are shown in Table 1. The Lysholm score improved from 55, preoperatively, to 100, 1-year postoperatively [6].

## 3. Discussion

Most cases of hypermobile meniscus reported in the past showed excessive hypermobility of the posterior part of the lateral meniscus [1–4]. The cause of hypermobility in the posterior part of the lateral meniscus was thought to be posttraumatic disruption of the popliteo-meniscal fascicles near the popliteal tendon [7]. This case had general joint laxity. However, to our knowledge, there was no report describing the relationship between general joint laxity and hypermobile meniscus. Additionally, hypermobility of a discoid meniscus has been well-reported [8–10]. Peripheral rim instability of the anterior and posterior horns of a discoid lateral meniscus has also been reported [11]. Furthermore, there has been a report on the hypermobile medial meniscus [12, 13]. In this report, we have presented a rare case of the hypermobile anterior horn of the lateral meniscus, which was not a discoid meniscus. To our knowledge, this is the first such reported case. Thus, we think it is important to investigate and evaluate the meniscus carefully to avoid overlooking the possibility of a hypermobile meniscus even in cases without meniscus tear on MRI.

Several treatment methods for hypermobile lateral meniscus have been reported in the literature. Kimura et al. reported good outcomes of meniscectomy [1]. They described that subtotal meniscectomy showed significantly higher scores than partial meniscectomy. Ohtoshi et al. reported the outcomes of arthroscopic thermal shrinkage [14]. They reported that four out of five patients had no recurrence of locking after surgery, and thermal shrinkage could be considered an appropriate treatment for hypermobile lateral meniscus. Recent studies have shown good outcomes of meniscus repair for hypermobile lateral meniscus. Steyn et al. reported the clinical outcomes for hypermobile lateral meniscus repair in 12 patients [3]; seven of the 10 patients did not report a recurrence of mechanical symptoms at the time of follow-up. Kamiya et al. [4] reported good clinical outcomes for hypermobile lateral meniscus repair using the inside-out technique. They reported that meniscus repair was performed in 20 patients, and the Lysholm knee scores significantly improved from 72.0, preoperatively, to 97.8, 2 years postoperatively. There were no complications or recurrences of locking symptoms at the final follow-up. Steinbacher et al. [15] also reported good clinical outcomes of meniscus fixation for a hypermobile lateral meniscus in 45 soccer players (46 knees); the postoperative International Knee Documentation Committee score was 86.2, and the postoperative visual analog scale score was 1. Three of the 46 cases required a reoperation. Recent studies reported that meniscus repair showed significantly better outcomes than meniscectomy for meniscus tears [16, 17]. Stein et al. [16] reported that for isolated meniscus tears, as compared to partial meniscectomy, arthroscopic meniscus repair showed significantly improved results regarding the long-term follow-up in osteoarthritis prophylaxis and sports activity recovery; no osteoarthritis progress was noted in 80.8% cases with meniscus repair versus 40.0% cases with partial meniscectomy (*P* = 0.005). Persson et al. [17] reported a 25-50% lower risk of consultation for knee osteoarthritis after meniscal repair than after partial meniscectomy. The number of cases undergoing meniscus repair as compared to meniscectomy has been increasing in many countries [18–22]. Thus, we decided to perform meniscus repair in our case and observed good short-term outcomes.

## 4. Conclusions

Careful arthroscopic evaluation is essential for the diagnosis of a hypermobile anterior horn of the lateral meniscus. Arthroscopic meniscus stabilization provides good short-term outcomes for hypermobile meniscus.

## Figures and Tables

**Table 1:** Pre- and postoperative physical examination in hypermobile anterior horn of the lateral meniscus.

	Preoperative	Postoperative, 1-year
ROM extension (°)	-10	0
Flexion (°)	130	145
Tenderness	Lateral joint space	None
McMurray test	Positive	Negative
Lysholm score	55	100

ROM: range of motion.
